# Medial Patellofemoral Ligament Reconstruction vs Nonoperative Treatment for Recurrent Lateral Patellar Dislocation: Three-Year Results From a Randomized Controlled Trial

**DOI:** 10.1177/03635465261416931

**Published:** 2026-02-08

**Authors:** Truls Martin Straume-Næsheim, Per-Henrik Randsborg, Tina Løkken Nilsgård, Asbjørn Årøen

**Affiliations:** † Department of Orthopaedic Surgery, Akershus University Hospital, Lørenskog, Norway; ‡ Oslo Sports Trauma Research Center, Department of Sports Medicine, Norwegian School of Sport Sciences, Oslo, Norway; § Faculty of Health Sciences, Oslo Metropolitan University, Oslo, Norway; Investigation performed at the Department of Orthopaedic Surgery, Akershus University Hospital, Lørenskog, Norway

**Keywords:** knee, patellar dislocation, knee ligaments, medial patellofemoral ligament, pediatric sports medicine

## Abstract

**Background:**

Medial patellofemoral ligament reconstruction (MPFL-R) is the primary surgical intervention for recurrent lateral patellar dislocation (LPD). Isolated MPFL-R is recommended for patients without anatomic high-risk factors that predispose to further dislocations.

**Purpose:**

To compare instability recurrence in patients with recurrent LPD without underlying anatomic risk factors treated with MPFL-R versus active rehabilitation.

**Study Design:**

Randomized controlled clinical trial; Level of evidence, 1.

**Methods:**

Patients aged 12 to 30 years with recurrent LPD and no underlying anatomic high-risk factors for further dislocations—specifically, no severe trochlear dysplasia (Dejour D) and a tibial tuberosity–trochlear groove distance ≤20 mm on computed tomography—were randomized to receive knee arthroscopy with isolated MPFL-R followed by active rehabilitation (MPFL group) or knee arthroscopy without reconstruction followed by active rehabilitation (control group). The primary outcome was subjective persistent patellar instability at 3 years. Knee function at baseline and 1 and 3 years was assessed by the following patient-reported outcome measure (PROM) scores: Knee injury and Osteoarthritis Outcome Score (KOOS), Kujala Knee Score, Cincinnati Knee Rating System, and Noyes Sports Activity Rating Scale.

**Results:**

Between 2010 and 2019, 61 patients (72.1% female) were included in the study and randomized, with 30 assigned to the MPFL group and 31 to the control group. At 3-year follow-up, subjective persistent patellar instability was reported by 5 patients in the MPFL group (16.7%) versus 15 patients in the control group (53.6%), corresponding to an odds ratio of 5.8 (95% CI, 1.7-19.4; *P* = .003). Both groups reported significant improvements in all PROM scores from baseline to 3 years. However, no significant differences in PROM scores were observed between the groups at any follow-up time point.

**Conclusion:**

Isolated MPFL-R was more effective than active rehabilitation alone in preventing patellar instability after 3 years.

**Trial Registration:**

ClinicalTrials.org (NCT02263807).

Recurrent lateral patellar dislocations (LPDs) have a high incidence among teenagers and young adults. This condition has the potential to be disabling, affecting not only participation in sports but also daily activities and overall quality of life.^11^ Isolated reconstruction of the MPFL (MPFL-R) has emerged as the prevailing stabilizing surgical intervention, with numerous studies documenting favorable outcomes after this single procedure across diverse patient populations.^8,22,23^ Current recommendations for isolated MPFL-R are recurrent LPD episodes in patients where bony procedures are contraindicated because of open growth plates or where there are no underlying anatomic high-risk factors for further patellar dislocations.^6,23,30^ The same anatomic criteria have been used to identify patients suitable for nonoperative management after a first-time patellar dislocation.^1,9^

Approximately 40% of patients with recurrent LPD fall into this category, characterized by the absence of severe trochlear dysplasia, patella alta, and a tibial tuberosity–trochlear groove distance <20 mm on computed tomography (CT) or <15 mm on magnetic resonance imaging (MRI).^26,27^ In the absence of significant bony anatomic risk factors, the primary focus for restoring patellar stability shifts to the soft tissue structures, particularly the MPFL and the vastus medialis oblique (VMO) muscle.^12,17,31^ The VMO, being a trainable muscle, plays a crucial role in dynamic stabilization of the patella. It could be argued that the favorable outcomes observed after isolated MPFL-R are, at least in part, attributable to the active postoperative rehabilitation focusing on strengthening the VMO, thereby enhancing patellofemoral stability through improved neuromuscular control. These considerations formed the rationale for the present randomized controlled trial (RCT), which compared isolated MPFL-R followed by active rehabilitation versus active rehabilitation alone in patients with recurrent LPD and no substantial anatomic risk factors.^27^

The 1-year result from this RCT is previously reported.^27^ After 1 year, the nonreconstructed cohort exhibited a 6-fold elevated risk of subjective persistent patellar instability. However, no statistically significant differences were observed between the groups regarding activity level or any patient-reported outcome measure (PROM) scores. The primary objective of the present study was to determine whether the difference in subjective persistent patellar instability between the treatment groups remained significant at the 3-year follow-up. A secondary objective was to evaluate whether there were any differences between the groups in knee function and PROM scores over time.

## Methods

### Participants

This study was a 3-year follow-up from an RCT that compared isolated MPFL-R followed by active rehabilitation with active rehabilitation alone in patients with recurrent LPD.^27^ The study included patients aged 12 to 30 years who had sustained ≥2 episodes of unilateral LPDs, with no severe trochlear dysplasia (Dejour D) and a tibial tuberosity–trochlear groove distance ≤20 mm on CT. These criteria were established to confirm that patients met the requirements for treatment with isolated MPFL-R. Before enrollment, all knees were examined by MRI to rule out cartilage injuries or other significant ligament injuries.

### Intervention

At the end of diagnostic knee arthroscopy, patients were assigned by block randomization (blocks of 10) to receive MPFL-R combined with active rehabilitation (MPFL group) or active rehabilitation alone (control group). We performed a modified all–soft tissue MPFL-R procedure using a semitendinosus autograft that remained intact at the tibial insertion. The graft was slung around the adductor tendon and secured with a basketweave fixation in the periosteum of the patella.^4,27^ Full straight-leg weightbearing was permitted from the first day postsurgery, and passive range of motion was allowed from 0° to 90° of flexion. Eight weeks later, patients in the MPFL group followed the same rehabilitation program as the control group. Patients in both groups were referred to primary care physical therapists, but one from our orthopaedic department instructed the patients at the day of surgery and conducted follow-up at 3 and 6 months. Return to sports was permitted after 6 months. The randomization process, surgical procedure, and rehabilitation details have been published.^27^

### Outcome Measures

At baseline, 1 year, and 3 years, patients were examined and interviewed to assess subjective persistent patellar instability. They also completed the Knee injury and Osteoarthritis Outcome Score^21^ (KOOS), the Cincinnati Knee Rating System,^20^ the Kujala Knee Score,^13^ and the Noyes Sports Activity Rating Scale.^20^ The activity rating scale was dichotomized into pivoting sports or nonpivoting sports. The KOOS subscales used in the analysis were sport and recreational activities (KOOS Sport) and knee-related quality of life (KOOS QoL), as they have been proven most sensitive to this young patient group.^28^

### Statistics

Power calculations were based on the observed 25% discrepancy in redislocation rate between the MPFL group and the control group. The number of patients required in each group was 28, given a power of 80% and a significance level of .05.^27^ Binary comparisons were assessed with the Pearson chi-square test. Odds ratios (ORs) were calculated by a logistic regression model adjusted for potential confounding factors. Independent or paired *t* tests were used to assess normally distributed data. All data were analyzed in SPSS Version 29 (IBM Corp).

### Ethics

The study was registered at ClinicalTrials.org (NCT0226 3807) and approved by the Regional Committee of Medical and Health Research Ethics of South-East Norway before its initiation (REC South-East, reference 2009/2148). All patients, including legal guardians (if patient age <18 years at the time of enrollment), provided oral and written consent before inclusion.

## Results

### Participants

**Figure 1.**
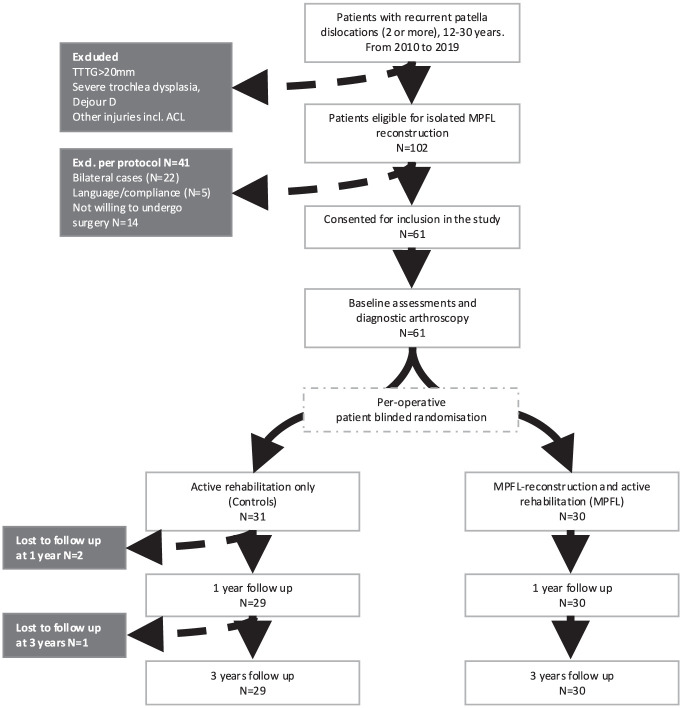
Flowchart of patient selection, randomization, and follow-up. ACL, anterior cruciate ligament; MPFL, medial patellofemoral ligament; TT-TG, tibial tuberosity–trochlear groove.

Between 2010 and 2019, 228 patients were referred to our institution for recurrent LPD. Of these, 80 (35.1%) with unilateral recurrent LPD met the inclusion criteria for isolated MPFL-R. Fourteen patients declined participation, and 5 were excluded for language barriers, resulting in 61 patients who provided informed consent and underwent perioperative randomization.^27^ These patients were assigned to either MPFL-R with active rehabilitation (MPFL group, n = 30) or diagnostic arthroscopy and active rehabilitation (control group, n = 31). Three patients (9.6%) in the control group did not attend the final follow-up at 3 years; there was no loss of follow-up in the MPFL group (Figure 1). Baseline demographics for all patients are presented in Table 1. One patient in the control group developed complex regional pain syndrome in the thigh, most likely related to tourniquet use during diagnostic arthroscopy. No infections or other significant postoperative complications were reported.

**Table 1:** Descriptive Comparison of the MPFL and Control Groups at Baseline*^*a*^*

	No. (%) or Mean ± SD	
	MPFL (n = 30)	Control (n = 31)
Female	22 (73.3)	22 (71.0)
Age, y	18.3 ± 4.9	19.9 ± 5.5
Age <16 y at baseline	11 (36.7)	11 (35.5)
Right knee	16 (53.3)	16 (51.6)
Body mass index, kg/m^2^	21.9 ± 3.3	25.3 ± 5.1
Patella alta*^*b*^*	5 (16.7)	5 (16.1)
Patellar instability severity score ≥4*^*c*^*	4 (13.3)	5 (16.1)

a MPFL, medial patellofemoral ligament.

b Patella alta defined as patellotrochlear index <0.25.^2^

c Patellar instability severity score by Balcarek et al.^1^ A score ≥4 is defined as high risk of new dislocation after a primary lateral patellar dislocation.

Arthroscopy revealed 31 minor cartilage injuries in 24 knees (39.3%). Of these, 25 (80.6%) were on the patellar surface, and debridement was performed in 5 (16.1%). No other significant intra-articular injuries were found.

### Subjective Persistent Patellar Instability

After 1 year, 2 patients (6.7%) in the MPFL group experienced subjective persistent patellar instability versus 13 controls (43.3%; *P* = .005). After 3 years, subjective persistent patellar instability was reported by 5 patients (16.7%) in the MPFL group and 15 (53.6%) in the control group, corresponding to an OR of 5.8 for the control group (95% CI, 1.7-19.4; *P* = .003). Of the 5 patients in the MPFL group with redislocations, 3 were female and <16 years old at the time of surgery. When correcting for age <16 years (yes/no), body mass index, and sex, the OR increased to 6.1 (95% CI, 1.5-24.7; *P* = .012). A sensitivity analysis showed that, even if all 3 control patients lost to follow-up remained stable at 3 years, the control group still had higher odds of persistent patellar instability (OR, 4.7; 95% CI, 1.4-15.4; *P* = .008).

### Symptoms and Activity

**Figure 2.**
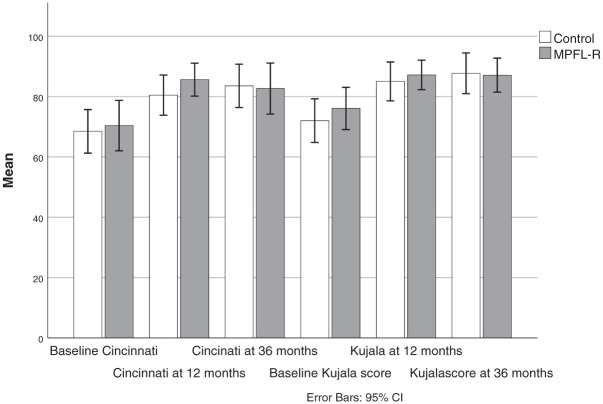
Cincinnati and Kujala total scores at baseline and 1- and 3-year follow-up for the control and MPFL groups. No significant between-group differences were found at any time points (independent samples *t* tests). Both groups improved from baseline to 12-month follow-up (paired samples *t* tests; *P* < .005) but not from 12 to 36 months. MPFL-R, medial patellofemoral ligament reconstruction.

**Figure 3.**
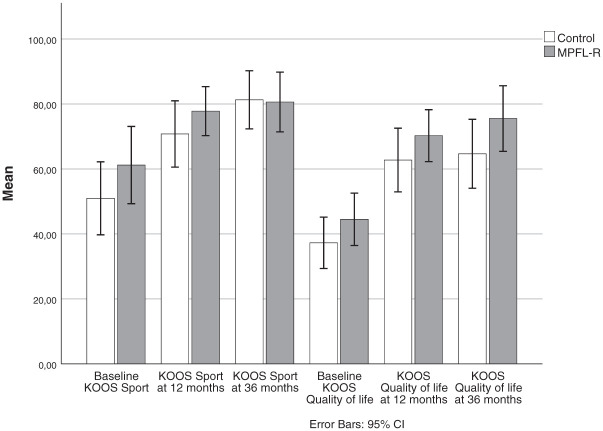
KOOS Sport and Quality of Life scores at baseline and 12- and 36-month follow-up for the control and MPFL groups. No significant between-group differences were found at any time points (independent samples *t* tests). Both groups improved from baseline to 12-month follow-up (paired samples *t* tests; *P* < .005) but not from 12 to 36 months except for the control group for KOOS Sport (*P* = .022). KOOS, Knee injury and Osteoarthritis Outcome Score; MPFL-R, medial patellofemoral ligament reconstruction.

Both groups demonstrated significant and clinically meaningful improvements in all 4 PROM scores from baseline to 3-year follow-up (Figures 2 and 3). The majority of these improvements occurred within the first year, as previously reported.^27^ Substantial improvement from 1 to 3 years was not found, except for KOOS Sport in the control group. There were no significant differences in any PROM scores between the groups at any follow-up time point.

**Figure 4.**
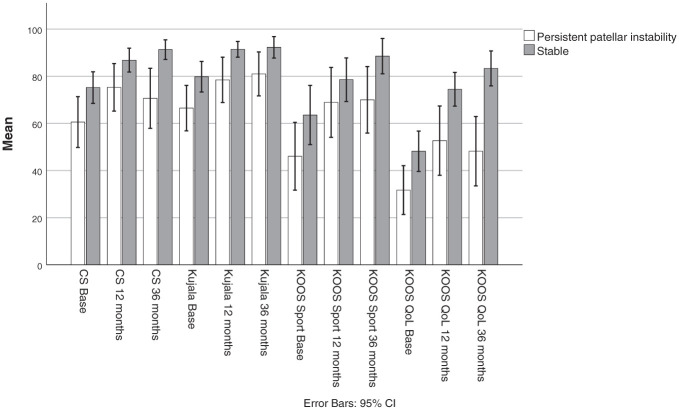
Comparison of all PROM scores at each follow-up point between patients (n = 20) who reported subjective persistent patellar instability at 3 years and their peers who were stable (n = 38), regardless of treatment group. Patients who were stable reported significantly higher PROM scores at all time points except for KOOS Sport at baseline (*P* = .067) and at 12 months (*P* = .068). CS, Cincinnati Knee Rating System; KOOS, Knee injury and Osteoarthritis Outcome Score; PROM, patient-reported outcome measure; QoL, quality of life.

When patients who were stable were compared with those reporting subjective persistent patellar instability at 3-year follow-up, the latter group consistently reported significantly lower scores across all PROMs at all time points regardless of intervention (Figure 4).

At baseline, 29 (47.5%) participants were actively engaged in pivoting sports. All patients returned to sport between 6 and 12 months postoperatively. After 3 years, this number was 23 (37.7%), with 10 in the control group and 13 in the MPFL group, and this was not significant (OR, 1.6; 95% CI, 0.6-4.6; *P* = .37). Among patients with subjective persistent patellar instability, only 4 (20%) remained active in pivoting sports as opposed to 19 (50%) who were stable (OR, 4.0; 95% CI, 1.1-14.2; *P* = .026), regardless of treatment.

## Discussion

In summary, isolated MPFL-R was significantly more effective in preventing further patellar instability as compared with active rehabilitation alone.

These results demonstrate that even in the absence of underlying high-risk anatomic factors, patients with ≥2 previous episodes of LPDs benefit from surgical treatment to prevent further dislocations. Persistent patellar instability significantly affects knee function and activities of daily living.^11^ Therefore, while anatomic risk factors should guide surgical technique, the decision to recommend surgery should be based more on clinical history than anatomy alone.

Between the 1- and 3-year follow-up, additional recurrent events occurred in both groups (3 in MPFL and 2 in control). In the MPFL group, the number of patients with recurrent dislocations more than doubled, from 2 at 1 year to 5 at 3 years, whereas the control group increased only slightly, from 13 to 15. Patients treated nonoperatively recover quickly, and the lack of a functional MPFL becomes apparent sooner. In contrast, patients undergoing MPFL-R may not have fully returned to pivoting sports by 1 year, leading to additional reported instability between years 1 and 3. Therefore, additional surveillance after MPFL-R is required.

Active rehabilitation alone can improve patient-reported knee function, even in patients with ≥2 patellar dislocations. Moreover, 14 (48.3%) of these patients remained stable without surgery. This is important knowledge for the patient and the surgeon. Our previous findings indicate that individuals with better preoperative performance on hop tests for distance are less likely to experience subjective persistent patellar instability.^19^ In the current study, we observed that patients who were stable also reported higher preoperative PROM scores. These findings highlight the importance of evaluating patients beyond anatomic risk factors, emphasizing the role of functional status and patient-reported outcome in the shared decision-making process with patients and parents before arriving at the appropriate treatment strategy for the individual patient. Caution should be taken in the interpretation of this secondary finding of no difference between treatment groups in PROMs, as our study was not powered for this

MPFL-R has been shown to be a safe procedure with a low complication rate.^3,18^ The most significant complications are typically related to malposition of the femoral fixation points and, less commonly, patellar fractures.^25^ The risk of these 2 complications is minimized with our all–soft tissue MPFL-R technique. However, according to our results and a recent review, the redislocation rate seems to be higher for the dynamic all–soft tissue MPFL-R technique than for fixed MPFL-R techniques.^7,23^ Even in a study in which isolated MPFL-R was used regardless of the bony anatomy, the redislocation rate was only 5%.^5^ This suggests that, for patients with a closed growth plate at least, a fixed MPFL-R technique with screw fixation in the femur should be considered. Many patients with recurrent LPD are adolescents, either still growing or just past the skeletal growth spurt but still physically developing. It is essential that these patients be informed of the risk of recurrent dislocation and the option of active rehabilitation. This allows them, with their parents or guardians, to engage in a shared and informed decision regarding the most appropriate course of treatment.

Active rehabilitation alone had a positive effect on the patients with recurrent LPD, as evidenced by similar improvements in PROM scores for both intervention groups. During the first year, follow-up occurred after 3, 6, and 12 months. Close follow-up is known to have a positive effect on PROM scores.^16^ However, there was no follow-up between 1 and 3 years. Therefore, the results at 3 years may better represent the daily knee function in patients with subjective persistent patellar dislocation. A recent qualitative study of patients with LPD found that many rely on fear-based avoidance strategies in their daily activities.^11^ While patients experiencing redislocations may initially adapt and compensate in a structured study setting, they often report a gradual decline in knee confidence when left to manage on their own. This trend was evident among patients reporting subjective persistent patellar instability at 3 years: their PROM scores plateaued or declined between years 1 and 3 in contrast to their stable peers, who continued to improve (Figure 4). Notably, patients who were unstable at 3 years reported lower PROM scores not only at 1 year but also at baseline. This suggests that PROM scores and physical performance tests may help identify patients at higher risk for unfavorable outcomes. Conversely, high PROM scores at baseline may indicate that some patients are coping well despite their LPD episodes, making the necessity of stabilizing surgery for this subgroup less apparent. Both aspects are valuable in guiding shared decision-making with the patient regarding the most appropriate treatment strategy.

Evidence on rehabilitation after LPD remains sparse. The existing literature emphasizes that rehabilitation should be individualized, phase-based, and progress according to functional criteria.^14,15,24,29^ Unfortunately, the collected data provided limited detail on the rehabilitation performed by primary care physical therapists. Since the initiation of the study, psychological factors have also been recognized as important for successful rehabilitation and return to sports and activities.^11^ The KOOS QoL score, which improved significantly across both groups, likely reflects these factors. However, the significant difference between patients who were stable and those with persistent patellar instability in KOOS QoL scores at 36 months demonstrates that this is more related to knee stability (Figure 4).

### Limitations

Of the PROM scores used in this study, only the Kujala Knee Score was designed primarily to assess patellofemoral disease. These PROM scores were the best available at the time when this study was designed, but they lack formal validation for this condition and study population.^10^ None of the included PROM scores contained questions specifically addressing persistent instability. As a result, this information was obtained through patient interviews and review of medical records, introducing a potential source of bias or the risk of missing relevant data.

Blinding to treatment was not possible given the nature of the intervention, and this could also affect the PROM scoring.

According to the original protocol, CT was used to identify the anatomic risk factors with the risk of underestimating some of the risk factors since. Accordingly, 20% of the included patients were identified as having a high risk of redislocation (patellar instability severity score ≥4; Table 1), when accounting for their age at first dislocation and their MRI findings. However, these were equally distributed between the groups. The small sample size also meant that further subgroup analyses were not possible. Nevertheless, the majority of patients with recurrent LPD in our study were characterized as having a low risk of redislocation. Yet, our patients had already sustained a redislocation despite having low anatomic risk factors after the primary event, placing them in a higher-risk category at inclusion. Therefore, our results cannot be generalized to the entire spectrum of patients with patellar instability.

Although surgery was not limited to a single surgeon, all procedures were performed at the same institution using the same all–soft tissue MPFL-R technique. This limits the external validity of our results, which cannot be directly extrapolated to other grafts and fixation methods.

Finally, we cannot draw firm conclusions about PROM differences between groups, as the study was not sufficiently powered for this secondary outcome.

## Conclusion

Isolated MPFL-R was more effective than active rehabilitation alone in preventing patellar instability after 3 years.
